# Disease in Mesopotamia

**Published:** 1919-12

**Authors:** F. P. Mackie


### DISEASE IN MESOPOTAMIA.

BY

Major F. P. Mackie, M.D., M.Sc., F.R.C.S., F.R.C.P., I.M.S.

---

In thanking the Society for the privilege of speaking on some of my experiences in Mesopotamia, I must apologise for the absence of figures and statistics from this paper. As all my records were left in Baghdad, I cannot trust my memory to supply accurate figures.

I left France for Mesopotamia at the end of 1915, and remained there till February, 1919. Until the spring of 1918 I was in Amara, in command of the Central Laboratory and Consulting Bacteriologist to the area. I was afterwards transferred to the newly-opened Central Laboratory in Baghdad, a much more ambitious affair, and there I remained nearly a year. The staff of this laboratory consisted of myself as O.C., an assistant bacteriologist, an analytical chemist, a protozoologist, an entomologist and a special malarial officer; whilst the laboratories of the Water Examination Unit with four chemists and the Veterinary Pathological Laboratories were housed under the same roof, and affiliated to the Central Laboratory. A mobile laboratory was formed, and stood ready with staff and equipment to start at a few hours' notice to investigate any outbreak of disease and to advise the local authorities on measures of prevention and cure. Of all pathological organisations in Mesopotamia the mobile laboratory was probably the most useful, and its work was certainly the most interesting.

I will now pass on to consider the chief points of interest about some of the principal diseases.

**Enteric Fever.**—This disease was prevalent throughout the campaign, and during 1916 especially it was one of the principal causes of severe illness and death.

*B. Typhosus* infections were much less common than those of *Paratyphoid A*. The figures from a total of 660 blood cultures in 1916 were 20 per cent. and 80 per cent. respectively. *Paratyphoid B.* was at this time excessively rare ; only twice was it recovered at the Central Laboratory, Amara, during the year. In following years the number of cases of *Enterica* were less, and the relative proportions of *B. Typhosus* and *B. Paratyphoid B.* increased as compared with *Paratyphoid A.* It was the practice to take blood cultures in all cases of fever lasting over four days (in which malarial parasites were not found), and up to the fourteenth day. Subsequent to that three different samples of the fæces and urine were examined. Clearing tests were also done on soldiers who were convalescent from enteric fever, and they were afterwards sent to India to carrier depots, where final clearing tests were done. This system of taking blood and stool cultures worked very well, and it is probable that few cases of enteric fever escaped undetected.

In the early days we attempted agglutination tests in those cases too late in the disease for blood culture ; but later on, when T.A.B. vaccination supplanted plain *typhosus* inoculation, this test was given up as unreliable, and eventually an order was circulated forbidding the diagnosis of these diseases by agglutination only. It is certain that no reliable conclusion can be come to by a single agglutination in an inoculated person, though it is possible to do so if a number of examinations are made at different periods throughout the disease and a curve of the reaction to all three organisms plotted and studied by an experienced observer. Professor Dreyer's method is very valuable in performing these quantitative tests.

During 1918 a strange paratyphoid organism appeared, which had probably come down from Western Persia and the region of the Caspian. This organism was recovered by blood culture from some twenty or thirty patients, and was first returned as an inagglutinable form of *Paratyphoid B.* It gave the sugar and morphological reactions of that organism, but failed to agglutinate with standard *Paratyphoid B.* high titre sera. Some strains, it is true, gave a modified or incomplete reaction with this serum, but most strains were not agglutinated by it at all. It was agglutinated in very high dilution by its own sera made by inoculating a rabbit, and a number of absorption and cross-agglutination tests showed it to be a specific organism and not merely a variant of *B. Paratyphoid B.* On these grounds we felt justified in taking the paratyphoids farther down the alphabet, and called it *B. Paratyphoid C.* It had previously been found at Erzingen in Turkey-in-Asia, and was there regarded as either *B. Aertrycke* or *B. Suipestifer,* but our investigations in Baghdad showed it to be different from both these organisms. From a clinical point of view it was interesting on account of its tendency to produce visceral complications, particularly pneumonia. It was recovered in a number of cases from the lungs in typhoid-pneumonia and also from other organs, also from abscesses following typhoid, and in one case from necrotic areas in the liver in an obscure typhoid-like disease. The occurrence of this organism has since been reported from other parts of the world.

Further details as to this organism can be found in a paper by myself and Captain Bowen, R.A.M.C., and another by Captain McAdam, R.A.M.C., in the *R.A.M.C. Journal* of August of this year.

**Dysentery.**—Both types of the disease were common, the amœbic type all the year round and the bacillary chiefly in the spring and autumn in epidemic form. An enormous amount of work in fæcal protozoology was done in my laboratory in Amara by Captain Lapage, R.A.M.C., but the subject is too involved and too controversial for me to say anything about it here. If an amœba was very active and contained red-blood cells, or if the fæces contained cysts with four or less than four nuclei, the evidence was accepted as being that of *E. Histolytica* and the disease being amœbic dysentery. Amœbæ and cysts not answering to this description were not returned as those of *E. Histolytica.* I am sceptical about most of the cases of flagellate dysentery. The only case in which I saw sufficiently large numbers of lamblia flagellates to justify a diagnosis of lamblia diarrhœa was proved on culture to be one of mild Asiatic cholera. *Balantidum Coli* undoubtedly produces diarrhœa, but it was very rarely found in Mesopotamia.

Amœbic abscess of the liver was frequently met with, and in many of the cases it quickly followed the disease in the bowel.

No attempt was made to isolate cyst carriers, as it was found that over 10 per cent. of apparently healthy soldiers carried histolytica cysts.

*Bacillary dysentery* was a much more formidable cause of invaliding and death. It showed a seasonal rise in the spring and autumn, especially the latter, and on several occasions it caused localised epidemics of great severity. There was a large refugee camp near Baghdad where some 30,000 refugees from the north-west parts of Persia and the region about Lake Van congregated. They marched down to escape the terrors of rapine, murder and starvation at the hands of the Turks, and those who did not die on the road were put in a large concentration camp. Dysentery made terrible havoc amongst these debilitated, half-starved women and children and many died.

The mobile section at the Baghdad Central Laboratory was sent up in charge of my assistant, Captain Bowen, and a collection of large intestines was sent down to us showing an almost unbelievable state of inflammation, ulceration, necrosis and sloughing. The large majority of these cases was due to *Shiga* infections, and often direct culture from the stools on MacConkey plates showed pure cultures of *Shiga* bacillus.

Just after the Armistice I took the mobile laboratory to Mosul to inquire into an undue prevalence of dysentery and diarrhœa. I found a variety of causes at work. One was the presence of saline aperient matter in the water, another the irritating quality of the flour taken over from the Turks; and another factor was the enormous number of flies and the fouling of all articles of food by them. Histolytica cysts were found in the alimentary canal of some flies and similar cysts on articles of food exposed in the bazaar.

**Cholera.**—There were several outbreaks of cholera, with only one of which, that at Amara in 1916, was I brought into close contact. This outbreak occurred in two British hospitals about the same time and resulted in a good many deaths. It was afterwards traced to contamination of milk brought in from Arab encampments, which had somehow escaped sterilisation at the hospitals. There was an epidemic in 1917 in Baghdad, of whom the most lamented victim was that great General, Sir Stanley Maude.

**Malaria.**—Mesopotamia as a whole is not badly infected with malaria. The rain comes in the cold weather, and by the time the temperature is high enough for the cycle to take place in the mosquito the water has dried up. This does not apply to Bursa or Kurna in the south, where flooding takes place from the swollen rivers in March and April. In the creeks and irrigation channels around the date palms *anopheles* breed in enormous numbers and malaria is prevalent.

When our troops were marching through Persia up to the Caspian they encountered very different conditions, and malaria became a perfect scourge.

In the summer of 1918 a series of cases of a malignant kind of jaundice occurred in some of the camps on the Persian lines of communication. Several British soldiers died, and others were invalided. I was sent up with my mobile laboratory in company with Colonel Willcox, the Consulting Physician to the Force, to investigate the disease, which was reported to be probably spirochætal jaundice. We found the disease to be a very severe form of malignant tertian malaria with jaundice, and this was verified by microscopic examination. Most of the cases were swarming with malignant tertian parasites, and several were infected with two or even three species of parasite. I was able to clinch the matter by finding anopheles in the tents, by finding their breeding-places near the camp, and finally by dissection of 100 consecutive insects found a *zygote* stomach infection in one and a sporozoit infection of the salivary glands in another. The local and only species of anopheles, *A. Nursei*, was thus added to the list of proved malarial carriers.

On another occasion I went with Major Christophers, the well-known malariologist, right up to the foot-hills of Persia opposite to Lake Van, some 300 miles north-east of Baghdad. There, amongst the most exquisite natural surroundings, we found a population more stricken with malaria than we had seen in the worst parts of India or Africa. Practically every child was diseased, and the results of profound anæmia and malnutrition were evident everywhere. We went up to choose a health resort for Baghdad, but we returned sadder and wiser men.

**Typhus and Relapsing Fevers** were met with during the cold weather, and were particularly virulent amongst the refugees who came down from Persia. There was no very marked epidemic of either disease amongst British or Indian soldiers, but labour corps and the civil population seemed to suffer more.

An epidemic occurred amongst the Turkish prisoners in the Busra concentration camp early in 1916, and the heavy mortality was attributed to malaria. I was sent down from Amara to investigate it. The epidemic was subsiding when I arrived, but there was enough evidence to show that the epidemic was a mixed one of typhus and relapsing fever—diseases of an identical epidemiology, being both louse-borne. The typhus and relapsing fever infections were brought down from Upper Mesopotamia by the Turks themselves. Relapsing fever in Busra at that time came from three distinct sources: one, the severest, was brought down by the Turkish prisoners; a second, much more mild, was brought in by a British regiment just arrived from Port Said; the third was an indigenous infection amongst the civil population in Busra bazaar.

The mortality amongst the Turkish prisoners had a political if not international importance, and the Government of India were very relieved to find, as my report showed, that the infection had been brought down by the prisoners themselves, and had not been contracted through any negligence on our part.

**Trench Fever.**—Another louse-borne disease, *Trench Fever,* was not noted in Mesopotamia. It is true that a distinguished member of this Society recorded his own case as probably one of trench fever. If it was so, it was the only case met with as far as I know in the campaign, though physicians were constantly on the look-out for it.

**Jaundice.**—There was on occasions a great prevalence of a simple catarrhal jaundice, such as was met with in the Dardanelles and on the Indian frontier, and which is called "camp jaundice." A very full and excellent account of these and other forms of jaundice will be found in the Lettsomian Lectures of this year by the Consulting Physician to the M.E. Forces, Colonel W. H. Willcox, C.B., C.M.G., A.M.S., in the *British Medical Journal* for May 10th, 1918, *et seq.* He deals fully with the types of jaundice noted in Mesopotamia, and briefly with the results of our investigations. Sufficient to say here that no infective agent was found in this camp jaundice, either by fæcal examination, blood examination, liver puncture, gall-bladder puncture, or by the removal of duodenal contents by Einhorn's sound. I employed all these measures without recovering any unusual organism.

The spirochætal type of jaundice was not found, though I carefully investigated five or six cases of jaundice of the acute yellow atrophy type, both before and after death, by dark ground illumination and by guinea-pig experiment, but never found a spirochæte. This fatal type of jaundice resembled those due to poisoning by tetra-chlor-ethane and other such drugs, and like those occasionally met with after salvarsan injections.

**Diphtheria.**—Bacteriological diphtheria was common, but clinical diphtheria very rare.

In one regiment I examined which had recently come from India about 30 per cent., I think, showed morphologically typical Klebs-Löeffler bacilli. They appeared to be non-toxic to the men, and were generally so to guinea-pigs. The organism persisted a very long time in the throat in spite of the most vigorous local treatment. In the case of one officer I remember he was kept in quarantine for 85 days, and his throat yielded an almost pure culture of diphtheria on every examination, but he never showed a symptom. This is the sort of case that causeth the enemies of bacteriology to blaspheme. Latterly we came to disregard diphtheria bacilli except under special circumstances.

**Heatstroke.**—Time does not permit me to say much about this disease, though there is much I might say. It claimed many victims, especially during 1916 and 1917, and I remember seeing 26 men admitted at once from one camp on a certain afternoon in July, 1916. They were all lying in rows in the entrance hall of one of the hospitals, whilst "bhistis" poured water over them.

It seemed that a man could resist very high temperatures as long as he remained in health; but if his temperature rose from whatever cause, such as sand-fly fever, malaria or paratyphoid, his heat-regulating centre seemed to "lose its head," and his temperature would soar up to 109° or 110° F.

I saw Sir Victor Horsley a great deal when he was out there. He often used to come into my laboratory and sit and talk over our work, and I had the sad duty of examining him in his last rapid illness and of seeing him to the end. He died of heatstroke, which developed rapidly, and he was unconscious the greater part of his illness, which only lasted about thirty hours.

In the investigation of heatstroke we used an instrument called the Kata thermometer, which I believe was introduced by Professor Leonard Hill, and found it very valuable in giving an indication as to the atmospheric conditions likely to be followed by heatstroke. It registers the rate of evaporation from a moist surface such as the human skin, and gives information which is not supplied either by the dry or wet bulb of an ordinary thermometer.

**Sand-fly Fever.**—This troublesome disease caused a great deal of inefficiency, especially during the hot weather and in standing camps. The fever only lasts three or four days, but those affected are left slack and depressed for a week or ten days afterwards, and some suffered from cardiac irritability or irregularity which interfered with a speedy return to active duties. It was especially prevalent in the standing camps at Busra, and almost everyone who passed through these camps during the height of the season fell a victim to this annoying complaint. Sand-flies were very numerous, and the only way to ensure protection was by the use of a very fine-meshed mosquito net, which many found almost unbearable by reason of its interference with ventilation at a time when the hot weather was at its worst. The causal organism of sand-fly fever has not been discovered, though it has been shown to exist in the blood of infected persons. It is almost certain that one or more species of sand-fly (*Phlebotomus*) is responsible for its transmission.

**Plague** appeared in epidemic form in all the three big towns, and the Central Laboratories at Busra, Amara, and Baghdad were responsible for the examination of all the rats trapped or found dead in the bazaars as well as for the diagnosis of human cases. As is usual, the rat epizootic preceded the human epidemic by a week or more, and thus some warning was given of the approaching human outbreak.

At one period in Amara in 1917 nearly half the rats found dead in the bazaar were found to be plague infected. Some weeks before the epizootic began on this occasion I received a mass of inflamed intestines and abdominal glands and pancreas which had been removed post-mortem from the body of a British soldier who had died from what was believed to be acute hemorrhagic pancreatitis. An operation for his relief had been performed, but had had to be abandoned on account of the condition found and because the man nearly died on the table.

I suspected at the time that the condition might be due to that rare disease primary abdominal plague, and was able to prove this by the isolation of *B. Pestis* from the lymph glands, which with the surrounding organs were in a state of the acutest hemorrhagic inflammation. A few days later another sporadic case was recognised by puncture of superficial femoral glands. It is an interesting epidemiological fact that such sporadic cases not infrequently precede the general outbreak, though of course they are often missed.

**Oriental Sore.**—This disease is extremely common in Baghdad, where almost every child in the bazaar shows a sore or the evidence of old infection. It was very common amongst both British and Indian soldiers. The better class Baghdadis artificially infect their children in some inconspicuous part of the body, and so avoid attacks on the face and subsequent disfigurement and scarring. Though the sores are often multiple when infected about the same time, one attack appears to confer immunity for the rest of life.

It is probable that this disease also is transmitted by sand-flies, but this has not been proved. When working in Assam on the relation of these flies to Kala-azar I found a proportion of the female flies were infected with an Herpetomonas parasite which is closely related to the genus *Leishmania.* It is interesting to note that sand-flies feed freely on the common wall lizard (ghecko), and these lizards are also infected with an Herpetomonas-Leishmanialike parasite. It has been suggested that Oriental sore, the sand-fly and the lizard stand in close relation the one to the other, but no opportunity presented itself of following out this idea, though there was plenty of all three of the factors in the houses of Baghdad.

Some discussion has recently taken place as to whether **Kala-azar** was contracted in Mesopotamia in those patients who developed it there. I had personal experience of most of the cases under discussion, and I take the view expressed by others that the evidence available is not sufficient to prove that in any of the cases the disease was actually contracted in Mesopotamia.

**Scurvy** was fairly common amongst Indian troops during 1916, when the operations for the relief of Kut held large numbers in an arid wilderness where fresh vegetables could not be obtained, and was particularly marked amongst non-meateaters. Those whose caste principles allowed them to eat meat (as well as British soldiers) did not suffer to the same extent. The disease showed itself chiefly in falling out of the teeth, swelling and ulceration of the gums, and extensive hemorrhages following trivial injuries or even spontaneously, especially in the legs. Some developed hemorrhagic joint effusions, whilst surgeons found that their operation wounds healed badly, broke down and suppurated frequently under circumstances where asepsis would ordinarily have rendered these conditions unknown.

**Beri-beri.**—This deficiency disease was met with from time to time both in British and Indian troops, and more frequently amongst the Chinese Labour Corps working at Busra. These latter men would only eat the highly-polished rice which had been completely deprived of the pericarp and germinal layers, and would not eat the locally-milled rice, which was coarse but contained the necessary vitamines. I think the difficulty was got over by giving them a more varied ration and by the issue of Marmite, which is a product of yeast and contains accessory food factors.

I did not see much of beri-beri from the laboratory point of view, though I remember an enthusiastic young medical officer rousing my interest in the matter by sending me a specimen with the following request, "Herewith specimen of fæces from a patient suspected of beri-beri for favour of examination for vitamines."

Before I close I wish to make a few remarks on the relationship of pathology to medicine which my experience in the investigation of disease in India has taught me, and which the late war has only served to confirm and emphasise.

I think our experiences during the war have taught us what advantages there are in the close co-operation between clinicians and pathologists, and I should like to see this principle extended still more into civil life.

The clinician will readily admit the great advantages of an early and exact diagnosis of the cases under his care, whilst the most cloistered pathologist must have felt the vitalising influence which close contact with the living patient must bring. Both will have learned that the sciences of pathology and medicine are not distinct, but are merely different aspects of one great problem—the prevention and cure of disease.

This co-operation has been to me something of an ideal, and one which I have tried to follow all my service ; and I should like to tell you, in this Society, from whom I first learnt the principle, and who in my opinion was one of its best exponents. It was my revered friend and teacher the late John Michell Clarke. He constantly taught the value of pathology at the bedside, and the advantages of bringing a vivid clinical experience to the laboratory and the post-mortem room.

I think it would be an advantage if pathologists visited the wards more, and so added their experience to that of the physician, to the greater advantage of the patient.

Again, the clinician might come more often to the laboratory, and there learn the results and the limitations of the various tests ; and he might at the same time take the pathologist more into his confidence by describing his cases, and by supplying charts and other documents to help him in his decisions. I think, too, that pathologists should be called in to investigate and advise in cases of dangerous or obscure diseases in private as well as in hospital practice.

The feeblest and least valuable of all forms of laboratory work is what one may call "long-range pathology." Here specimens, often badly collected, are sent a distance with a minimum of information. In such cases the laboratory is treated as a penny-in-the-slot machine, and little good can be expected from the result.

The routine work of a clinical laboratory is not, however, the most important or the most interesting that a pathologist has to do ; his greatest work is to discover the beginnings of disease, and to study the underlying processes of which the clinical symptoms are only a reflection.

It is through pathology that the greatest advances in medicine have been gained in recent years, whilst the sanitarian looks to its decision before he can make any real advance in Public Health.

All eyes are turned to the prevention of disease, and to the day when the best brains in the profession will be freed from the task of trying to cure individuals of those diseases which it is the aim of preventive medicine to prevent.

